# Modular Current Stimulation System for Pre-clinical Studies

**DOI:** 10.3389/fnins.2020.00408

**Published:** 2020-04-30

**Authors:** Soheil Mottaghi, Niloofar Afshari, Oliver Buchholz, Samuel Liebana, Ulrich G. Hofmann

**Affiliations:** ^1^Section for Neuroelectronic Systems, Department of Neurosurgery, Medical Center University of Freiburg, Freiburg, Germany; ^2^Faculty of Medicine, University of Freiburg, Freiburg, Germany; ^3^Technical Faculty, University of Freiburg, Freiburg, Germany; ^4^Department of Engineering, University of Cambridge, Cambridge, United Kingdom

**Keywords:** Modular current source, current stimulation, biphasic stimulation, deep brain stimulation, arbitrary waveform

## Abstract

Electric stimulators with precise and reliable outputs are an indispensable part of electrophysiological research. From single cells to deep brain or neuromuscular tissue, there are diverse targets for electrical stimulation. Even though commercial systems are available, we state the need for a low-cost, high precision, functional, and modular (hardware, firmware, and software) current stimulation system with the capacity to generate stable and complex waveforms for pre-clinical research. The system presented in this study is a USB controlled 4-channel modular current stimulator that can be expanded and generate biphasic arbitrary waveforms with 16-bit resolution, high temporal precision (μs), and passive charge balancing: the NES STiM (Neuro Electronic Systems Stimulator). We present a detailed description of the system’s structural design, the controlling software, reliability test, and the pre-clinical studies [deep brain stimulation (DBS) in hemi-PD rat model] in which it was utilized. The NES STiM has been tested with MacOS and Windows operating systems. Interfaces to MATLAB source codes are provided. The system is inexpensive, relatively easy to build and can be assembled quickly. We hope that the NES STiM will be used in a wide variety of neurological applications such as Functional Electrical Stimulation (FES), DBS and closed loop neurophysiological research.

## Introduction

Electrical stimulation was recommended in ancient Roman medical scriptures to treat severe headaches using the electric discharges of atlantic torpedo rays (Largus, 1983). Medically relevant beneficial electrical stimulation has since then, and particularly in the last few decades, come a very long way in the biomedical field, as well as in rehabilitation and sports medicine (Petrofsky and Phillips, 1984; Wu et al., 2002; Hamid and Hayek, 2008; Maffiuletti, 2010; Brinton et al., 2014; Bin Altaf et al., 2015). Today, electrical stimulation of the brain can achieve reliable mitigation of the symptoms of neurological diseases such as Parkinson’s disease (PD) or dystonia (Benabid et al., 2002; Tronnier et al., 2002; Vidailhet et al., 2005; Hardesty and Sackeim, 2007; de Hemptinne et al., 2015; Tronnier et al., 2015), can reduce chronic pain (Russo and Sheth, 2015), and reduce seizure incidents in epileptics (Velasco et al., 2000; Vonck et al., 2002). Most recently, advances regarding psychiatric disorders like obsessive compulsive disorder (OCD; Alonso et al., 2015) or major depression disorder (Schlaepfer et al., 2013) have also introduced electrical stimulation as an effective treatment.

In all of these applications, electrical stimulation is delivered as either current or voltage driven charge injection into brain tissue through small noble metal electrodes (Tehovnik, 2006; Rattay et al., 2012). In the case of voltage driven charge injection, the transferrable charge is sometimes hindered by a time-varying impedance of the interface between electrode and tissue (McConnell et al., 2009; Sooksood et al., 2010; Karumbaiah et al., 2013; Nag et al., 2013; Washburn et al., 2014; Ramirez De Noriega et al., 2015). Due to biotic factors such as tissue reaction, glial encapsulation at the electrode-tissue interface and electrochemical factors, voltage stimulation frequently needs to be performed regardless of this limitation (Biran et al., 2005; Gimsa et al., 2005). In contrast, current stimulation delivers the desired charge reliably over time but is inconvenienced by its more complex electronic setup (Nag et al., 2013; Washburn et al., 2014; Ramirez De Noriega et al., 2015).

In light of the growing interest in bioelectronic medicine, there is a need for user-friendly, affordable, and standalone yet precise stimulators coping with changing requirements in stimulation paradigms (Sahin and Tie, 2007; Jezernik et al., 2010; Wongsarnpigoon et al., 2010; Foutz and McIntyre, 2010; Schor and Nelson, 2019). These needs can only partially be satisfied by any of the multiple commercially available stimulation devices. Cost, proprietary firmware, dependence on electrophysiological recording setups, and companies’ policies can be prohibitive for customization and improvement research. Consequently, there are various custom-designed electrical stimulation systems which are tailored to the requirements of targets such as cardiac tissue (Tandon et al., 2011), cell cultures (Yuan and Silberstein, 2016), brain slices (Li et al., 2015), deep brain areas (Gong et al., 2015), and muscles (Wang et al., 2015; Stewart et al., 2016) in closed-loop and other electrophysiological applications (Sanders and Kepecs, 2014). In this paper, we introduce a low cost (see Appendix A for details) modular electrical current stimulation system that can be used in all of the above-mentioned applications. We hope to encourage researchers not to limit themselves to cloning the system, but to improve and develop it further. A thorough and detailed demonstration of the system’s elements, including links to the downloadable documents, is given in the Materials and Methods section. The implementation and characterization of the system, as well as its application, are presented in the Results section. Finally, we compare the Neuro Electronic Systems Stimulator (NES STiM) to two commercially available stimulators, presented in the Discussion.

## Materials and Methods

The NES STiM consists of four modular 16-bit current stimulation units, which can generate arbitrary biphasic current pulses. It is compatible with MacOS and Windows and can be used as a standalone unit. All the technical details of the system, from electronic schematics, printed circuit board (PCB) drawings, drivers, and firmware to software interfaces for MATLAB are available in our repository (Mottaghi et al., 2020). The instructions on how-to setup a NES STiM, are provided in the repository called (HowTo).

The NES STiM can be used in standalone or PC mode. In the standalone mode, all parameters should be predefined in the device and activate the output by external trigger. in PC mode all stimulation parameters can be defined by the MATLAB function or graphical user interface (GUI) before generating the output pulses.

**FIGURE 1:**
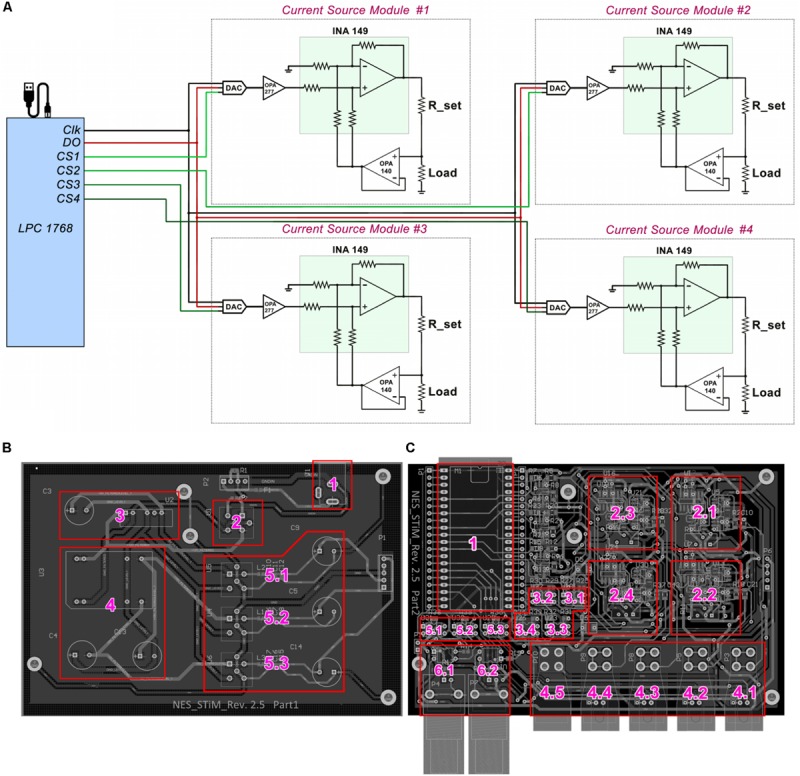
Schematic drawing of NES STiM system. **(A)** LPC1768 activates desired channels via their chip select (CS) pins, and places the SPI binary streams on the data bus (DO). With each clock signal, one bit is received by the DAC8831. **(B)** Essential units of the power unit are labled with numbers; 1) Input power socket receives +12 V power supply, which is filtered by the unit 2. The clean and filtered +12 V is fed to the units 3 and 4 in order to produce +5 and ±15 V, respectively. The generated +5 and ±15 V are then filtered by 5.1–5.3 and provided to the main board. **(C)** The input powers received from the power unit are only connected to the main board’s subunits only when the LPC1768 (unit 1) activates three ISO7000X optical switches (5.1–5.3), which is only done before the stimulation activation. Units 2.1–2.4 are the stimulation modules for corresponding to the channels 1–4, respectively. Charge balancing optical switches (3.1–3.4), current outputs for channes 1–4 (4.1–4.5), the ground connector (4.5), and digital input/output triggers (6.1–6.2) are labeled as well.

The NES STiM’s power unit provides low noise, medical standard ±15 V and 5 V outputs to be used by the main board subunits. TEL 3-2023 and TMA 1205D, two medical grade isolated DC/DC converters (Traco Electronic AG., Switzerland) were integrated to supply the required power. The essential components of the power unit are depicted in Figure 1B.

The processing module also regulates the power consumption of the system by monitoring the system’s state using optical switches IS7000X (ISOCOM, United States) (Appendix A) and connecting or disconnecting the power supply to the main board accordingly. The NES STiM features two BNC ports (one for the input trigger and one for the output) in case synchronization with other instruments is needed. Moreover, each channel has a specific LED, indicating whether the channel is active or not. Both port triggering and LED activation are also controlled by the processing unit (see Appendix A).

The mbed LPC1768, a prototyping module with a 32-Bit ARM Cortex-M3 microcontroller (NXP semiconductors, Netherlands), 30 input/output (I/O) ports and two integrated Serial-Peripheral-Interface (SPI) units, was selected for the processing module (see Appendix A). It benefits from a lightweight online C++ compiler and drag-*n*-drop programming which makes developing the system relatively easy. In PC mode, LPC1768 receives the desired parameters and start/stop commands via a mini USB-B port from the host PC. A serial port is assigned to the LPC1768 and all communications between the PC and NES STiM are conducted through this port (see the serial port setup procedure in the C code in the Supplementary Material).

The LPC 1768 transfers the stimulation parameters for each channel via SPI. The SPI data is placed on the data-bus (DO), but only the stimulation modules which have their chip-select pin (CS) activated, receive the data. 16-bit digital-to-analog DAC8831 converters receive the data as the first stage of the stimulation module and produce an amplified analog voltage between −2.5 V to +2.5 V (see Appendix A). The DAC’s analog output voltage is converted into current using a voltage controlled current source (modified Howland current pump) (Stitt, 1990) (see Figure 1A). Four pulse waveshapes [rectangle, sinusoidal, triangle, and linear decay (sawtooth)] are pre-defined in the C code of the LPC1768, which can be customized when needed. The stimulation pulses can be either generated for a defined number of pulses or continuously until the stop command is sent from the PC.

### Safety

To protect the tissue from excessive charge accumulation, a passive charge balancing mechanism was implemented (Sooksood et al., 2009; Sooksood et al., 2010). A 1 μF capacitor was mounted in series with the load (electrode) to prevent a net DC current, which could result in pH change and potential tissue damages. It has been shown that the charge density safe threshold for microelectrodes is between 100–200 μC cm^–2^ and around 30 μC cm^–2^ for macroelectrodes used clinically (McCreery et al., 1990). A warning pop-up window with the “USE AT YOUR OWN RISK” message appears when the user runs the GUI. Since the surface area of the electrode that the user utilizes determines the stimulation amplitude and pulse-width limits per phase, a highlighted note at the beginning of all the codes (MATLAB and C) is added as a warning before the experiment can be started. Additionally, the LPC1768 discharges the electrode potentials in interpulse intervals via activating optical switches (ISO7000x, ISOCOM, United States) (see Appendix A). The main subsections of the mainboard design are depicted in Figure 1C.

### Animal Experiments

Every procedure involving animal experiments was conducted in accordance with the guidelines of the German Council on Animal Protection. The protocols were approved by the Animal Care Committee of the University of Freiburg under the responsible supervision of the Regierungspräsidium Freiburg (approval G15/031) in accordance with the guidelines of the European Union Directive 2010/63/UE.

All the rodents, to be experimented on, were handled for several days in order to habituate to the new environment and experimenter. Female Sprague-Dawley rats (*n* = 21) underwent stereotactic surgery for unilateral 6-hydroxydopamine (6-OHDA) lesioning. They were anesthetized initially with 5% isoflurane and oxygen (0.15 l/min). Isoflurane concentration was lowered to 1.5% after fixing the animal in the stereotactic frame (David Kopf, United States). Animal reflexes, breathing and anesthesia depth were monitored throughout all the surgeries. Freshly prepared 6-OHDA neurotoxin solution (3.6 mg 6-OHDA dissolved in 1 ml of 20 mg ascorbic acid and 10 ml 0.9% NaCl solution) was prepared before each surgery and kept on ice and away from direct light throughout the surgical procedure (Sigma-Aldrich Chemie GmbH, Germany). 6-OHDA solution (3.3 μl) was administered using a microinjection pump UMP3 UltraMicroPump (World Precision Instruments, United States) either to the substantia nigra pars compacta (SNc; AP = −3.2 mm, ML = −1.5 mm from bregma, and DV = −7.2 mm from dura) with an injection speed of 0.5 μl/min or to the medial forebrain bundle (MFB; AP = −4.4, −4.0 mm, ML = −1.2, −0.8 mm from bregma, and DV = −7.8, −7.2 mm from dura) with an injection speed of 1 μl/min. The needle was left in the brain for 5 min after the injection to allow the brain to absorb the neurotoxin. The drill hole was filled with bone wax and the scalp then carefully stitched. Animals were given 14 days of recovery after lesioning.

All animals were tested using an Apomorphine test (Ungerstedt and Arbuthnott, 1970) in order to assess the success of the lesioning surgery. This test challenges the severity of dopamine depletion using a subcutaneous apomorphine solution injection (1 mg apomorphine, 2 mg ascorbic acid, 20 ml NaCl; 0.1 ml/100 gr of rat body weight, Sigma-Aldrich Chemie GmbH, Germany) to induce counter-clockwise rotations relative to the lesioned hemisphere. The rats showing an average of at least 3 counter-clockwise rotations per minute over a 30-min interval were categorized as the PD group.

In a separate stereotactic surgery, PD animals were implanted with stimulation electrodes positioned in the subthalamic nucleus (STN; AP = −3.6 mm, ML = −2.5 mm from bregma, and DV = −7.8 mm from dura) ipsilateral to the lesioned hemisphere. Bipolar stimulation electrodes consisting of two intertwining 50 μm coated Platinum/Iridium microwires (Science Products GmbH, Germany) were used in the course of this study. Stimulation electrodes with impedances <20 kΩ were selected for implantation. The animals were given 1 week of recovery after surgery.

### Accuracy and Reliability Assessment

To evaluate the precision of the NES STiM, several parameters relevant to neurophysiological applications were tested. These tests were all performed on a single NES StiM and on two computers with Windows and MacOS operating systems.

**FIGURE 2:**
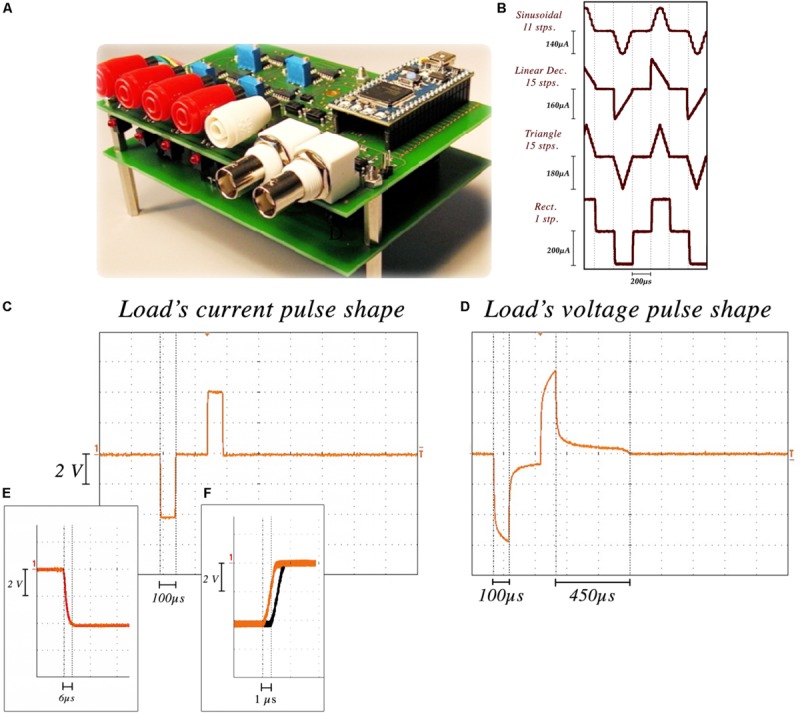
NES STiM characterization. **(A)** the NES STiM system. **(B)** Comparison between sinusoidal (11 steps), linear decreasing (15 steps), triangle (15 steps), and standard rectangular (1 step) pulses. **(C)** Current pulse measured from an 80 W resistor placed in series with the electrode. **(D)** Electrode potential of the same pulse, showing the duration of the passive charge balancing required for the potential to reach the pre-pulse value. **(E)** Rise and fall time of each step ≈6 μs. **(F)** The latency between first and last channel outputs.

The NES STiM’s reliability and precision characterization (Figures 2A,B) was performed using the aforementioned stimulation electrodes immersed in 0.9% saline. We performed 12-h tests which measured the current from a channel stimulating with a standard rectangular high frequency stimulation (HFS) waveform with 250 μA amplitude, 100 μs PW, 100 μs interphase interval, and 130 Hz frequency. The injected current was measured by the potential difference over an 80Ω resistor placed in series with the electrode. Due to the electrode tissue interface (ETI) and the capacitive characteristic of the ETI (Merrill et al., 2005), the electrode potential is smoothed (Figures 2C,D). The average rise and fall time for 100,000 pulses was 6.2 ± 1.3 μs. The passive charge balancing mechanism required 455 ± 32 μs, on average yielding a maximum tolerable frequency of 1.27 kHz.

Waveform shape was shown to impact the injected charge and energy-efficiency of the stimulation (Brocker and Grill, 2013). Digital arbitrary waveforms are composed of multiple discrete steps with different values and timings. Figure 2E shows four different waveforms (sinusoidal, linear decay, symmetric triangular, and rectangular) generated using this technique. Waveform resolution can be controlled by changing the number of discrete steps per phase. More steps in each phase results in a smoother waveform, while taking more time in total per phase. There is hence a trade-off between the minimum PW of a waveform and the number of steps in each phase (i.e., resolution). As an example, if we assume a single step requires 6 μs, 60 μs is the minimum time needed to produce a 10-step pulse.

Temporal latencies between channel outputs was another feature to test. Similar stimulation parameters were set for all channels and the delay between the outputs of the first and last channel was measured. As shown in Figure 2F, a 1.3 ± 0.18 μs delay was observed on average.

### Applications

High frequency DBS (>100 Hz) has been shown to be effective in treating movement disorders like those of PD patients. It alleviates motor symptoms such as tremor, rigidity, and akinesia (Lanotte et al., 2002; Brocker and Grill, 2013). The therapeutic frequency window is reported to be 100 Hz < *f* < 180 Hz with ceiling and floor limits of 250 Hz and 50 Hz, respectively (Moro et al., 2002).

**FIGURE 3:**
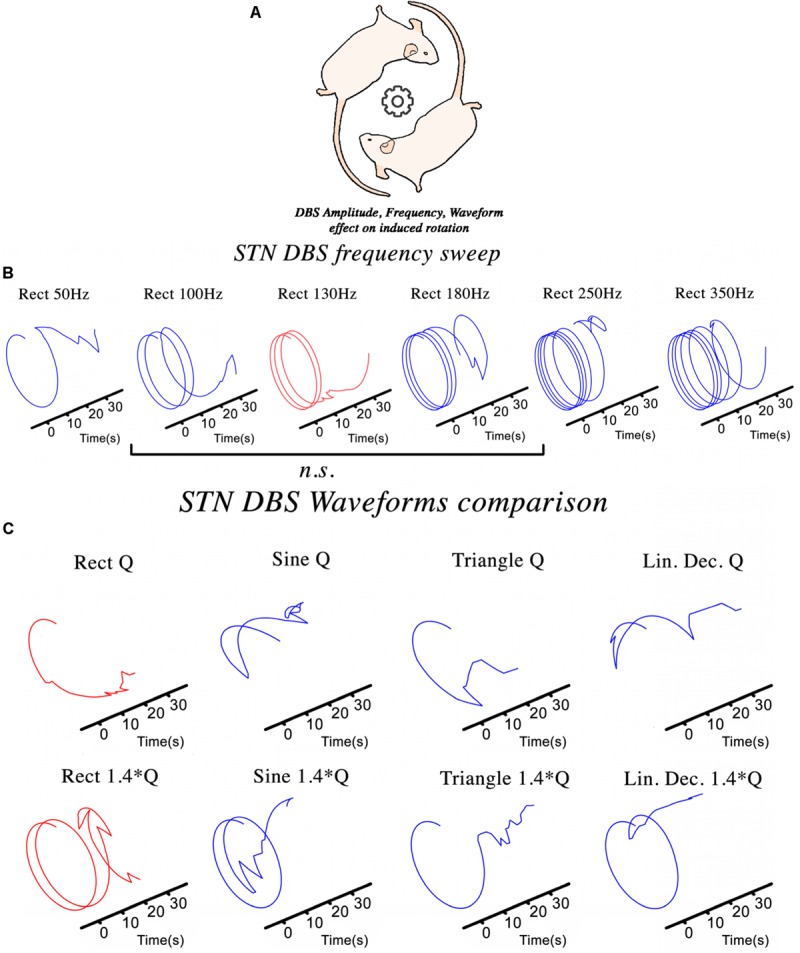
The NES STiM was utilized in different experimental studies using a hemi-PD rat model. **(A)** An assessment of the effect of STN-DBS parameters on the induced rotational behavior of the PD rats; **(B,C)** The impact of frequency and waveform on the induced rotation caused by stimulation.

The hemi-PD rat model is a well-established pre-clinical platform for testing novel stimulation paradigms not easily examined in patients. The NES STiM was used to apply four different waveforms (rectangular, sinusoidal, symmetric triangular, and linear decay) to hemi-PD rats and compare the induced contralateral rotation as in the frequency sweep test (Figure 3B). The evaluation of DBS in a hemi-PD rat model was the original reason for designing and developing the NES STiM. This device has been tested in various experimental paradigms related to the mentioned PD model. Examples are the impact of frequency and waveform on the effectiveness of DBS (see Figure 3), as well as implementing closed-loop DBS (Castaño-Candamil et al., 2017).

To test the effect of varying stimulation frequency on the hemi-PD rat model, we quantified the contralateral (to the lesion) rotational effect caused by biphasic rectangular electrical stimulation (So et al., 2017). Each animal was placed and habituated for >7 days in a large cornerless, semi-spherical bowl before being tested. DBS was then applied with increasing stimulation frequencies for 30 s at each value with a 45 s pause between different frequencies (see Figure 3B). Statistical evaluation of the observed rotational effect showed smaller Euclidean Distance (ED) for biphasic rectangular stimulation with frequencies between 100 and 180 Hz, whereas smaller (50 Hz) or higher stimulation frequencies (250 and 350 Hz) showed significantly higher ED values (ED_130__Hz.vs.100__–__180__Hz_ = 2.62 ± 0.22, ED_130__Hz.vs.__50__Hz_ = 4.08 ± 0.17 and ED_130__Hz.vs.__250__–__350__Hz_ = 12.89 ± 4.43).

Another essential aspect of electrical stimulation is the waveform. Studies on tissue damage (Yuen et al., 1981; McCreery et al., 1990; Shannon et al., 1995), power efficiency (Bin-Mahfoodh et al., 2003; Anheim et al., 2007), and DBS energy efficiency (Foutz and McIntyre, 2010; Brocker and Grill, 2013) are all valuable examples of the importance of waveform shape. We depict in Figure 3C the rotational effects induced by different waveforms, but comparable charge injections. Charge injection (Q) is normalized to the usual biphasic rectangular 130 Hz stimulation and alternated between the waveforms described in Figure 2E.

The history of closed-loop DBS goes back to (Osorio et al., 2001), where it aimed to control DBS by seizure detection. An invaluable closed-loop DBS study was performed using a primate model of PD (Rosin et al., 2011), which showed potential superiority over conventional open-loop DBS. This investigation inspired several studies assessing the method for human patients (Little et al., 2013, 2016). In these studies, beta oscillatory activities from local field potential (LFP) recordings were used to control the DBS. Consequently, the third test conducted using the NES STiM was a closed-loop DBS study on the hemi-PD rat model. In this study, beta band power was used to trigger the DBS in rats. Preliminary results from the study were published in (Castaño-Candamil et al., 2017).

## Discussion

Electrical stimulation and DBS studies in animal models will benefit from a robust, precise, yet modular electrical stimulation device augmenting available access to well-made commercial devices such as Plexon Stim (Plexon, United States) and AlphaSnR (AlphaOmega, Israel). Challenges in adjusting the latter to our research requirements motivated us to design and build the NES STiM.

In order to use the AlphaSnR stimulation device, a complex electrophysiological set-up has to be employed. This makes it rather prohibitive for a broad use and too expensive for many users. Plexon Stim can be used in standalone mode, but low compliance voltages make it difficult to stimulate using high impedance microelectrodes: output voltage reaches saturation fast and causes the output current to decline. A comparison of the specifications of the NES STiM and the two mentioned commercial devices is summarized in Table 1.

**TABLE 1:** Specification overview between the NES STiM and two commercially available stimulation devices (Plexon Stim, and AlphaLab SnR).

**Model name**	**NES STiM**	**Plexon Stim**	**AlphaLab SnR**
Output channels	4	16	8
Current modules	4	16	3
Output voltage	±13.5 V	±10 V	60 V
Polarity	Anod./ Cathod. First	Anod./ Cathod. First	Anod./ Cathod. First
Output current	1 μA–2500 μA, 1 μA increment	1 μA–1000 μA 1 μA increment	2 μA–3500 μA
Stimulation frequency	0.005 Hz–25 kHz	0.008 Hz–100 kHz	1–300 Hz
Pulse width	40 μS–65535 μs	5 μS–65535 μs	10–1000 μs
Inter-phase intervals	2 μS–Inf μs	5 μS–65535 μs	0–1000 μs
PC hardware interface	Mini USB B	Mini USB B	Ethernet
Stim manager PC Software compatibility	Windows Mac	Windows	Windows
API	Matlab, C++	C/C++ and Matlab	X86 / x64 library version
Analog resolution	16 bits	16 bits	16 bits
Dependency	–	–	AlphaSNR

An important difficulty concerns any customization which may be required for specific experimental paradigms. Since the technical designs of the commercial devices are not public, if at all possible, it would be cumbersome to arrange these customizations relying on the technical support from these companies. For instance, eventhough the NES STiM has 4 channels, however, the number of channels can be expanded by adding stimulation modules. In the current setup, in addition to the shared DO and Clk pins, each channel needs a CS and two pins for LED and charge balancing switch (one for each). LPC1768 has nine unused I/O ports which can support another 3 channels (7 channels in total). If more channels are still needed, replacing the LPC1768 by more capable products such as STM32F429I-DISC1 that contains 144 I/O ports could be an alternative.

Another customization for the NES STiM, is to add the bootstrapping mechanism explained (Mottaghi et al., 2015) to increase the compliance voltage to a desired level, while keeping rest of the components as they are in NES STiM. In order to challenge the device and high compliance voltages, up to 2000 μC cm^–2^ charge density was tested in flexible microelectrodes (Mottaghi et al., 2015). The NES STiM’s design is instead modular and it has been used as a stimulation device in a variety of experiments successfully. Details of said experiments will be published elsewhere and exceed the scope of this presentation of our modular NES STiM. We hope that this device will be cloned, customized and improved by other groups, engineers and researchers.

## Conclusion

Neuro Electronic Systems Stimulator is a modular electrical stimulation system for electrophysiological applications. The system has four channels, with a dedicated current source for each channel. It can be controlled from the PC via a USB connection or operate in standalone mode. Schematics and drawings of the electronics are available online together with the MATLAB and C++ control programs. Although stimulation parameters such as amplitude, frequency, pulse shape, and pulse width can be actively selected NES STiM does fit in a closed loop stimulation experiment as well (Castaño-Candamil et al., 2017).

## Ethics Statement

The animal study was reviewed and approved by University medical Freiburg, G15/031.

## Author Contributions

SM, NA, and UH contributed conception and design of the study. SM organized the work. SM performed the precision tests. SM wrote the first draft of the manuscript. SM, SL, OB, and UH wrote sections of the manuscript. All authors contributed to manuscript revision, read and approved the submitted version.

## Conflict of Interest

The authors declare that the research was conducted in the absence of any commercial or financial relationships that could be construed as a potential conflict of interest.
