# Supplementary material for: Modular Current Stimulation System for Pre-clinical Studies
Source: Front Neurosci. 2020 Apr 30;14:408. doi: 10.3389/fnins.2020.00408 (PMC7203490; doi:10.3389/fnins.2020.00408)
Supplement: Supplementary file 1 [file Data_Sheet_1.pdf]

Power Unit

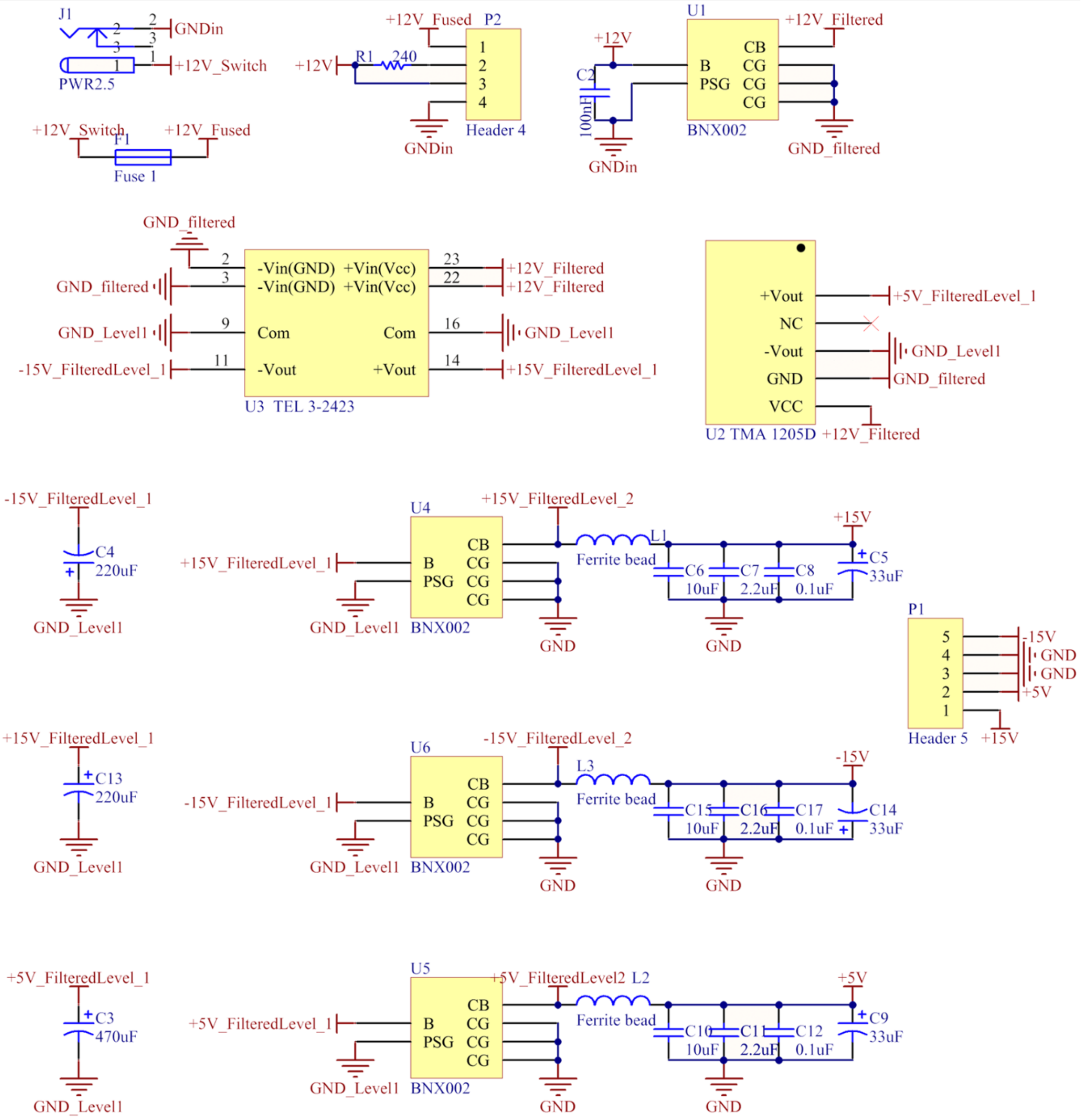
J1
GNDin
+12V_Switch
PWR2.5
+12V_Switch
+12V_Fused
F1
Fuse 1
+12V_Fused
P2
+12V
R1
240
Header 4
GNDin
U1
+12V
C2
100nF
CB
B
CG
PSG
CG
CG
BNX002
+12V_Filtered
GND_filtered
GNDin
GND_filtered
-Vin(GND)
+Vin(Vcc)
Com
-Vout
+Vout
U3 TEL 3-2423
GND_Level1
-15V_FilteredLevel_1
+12V_Filtered
+15V_FilteredLevel_1
+Vout
NC
-Vout
GND
VCC
U2 TMA 1205D
+5V_FilteredLevel_1
-15V_FilteredLevel_1
C4
220uF
U4
+15V_FilteredLevel_2
L1
Ferrite bead
+15V
C5
33uF
C6
10uF
C7
2.2uF
C8
0.1uF
BNX002
GND
P1
-15V
+5V
+15V
Header 5
+15V_FilteredLevel_1
C13
U6
-15V_FilteredLevel_2
L3
-15V
C15
C16
C17
C14
+5V_FilteredLevel_1
C3
470uF
U5
+5V_FilteredLevel2
L2
+5V
C10
C11
C12
C9

Processing Unit

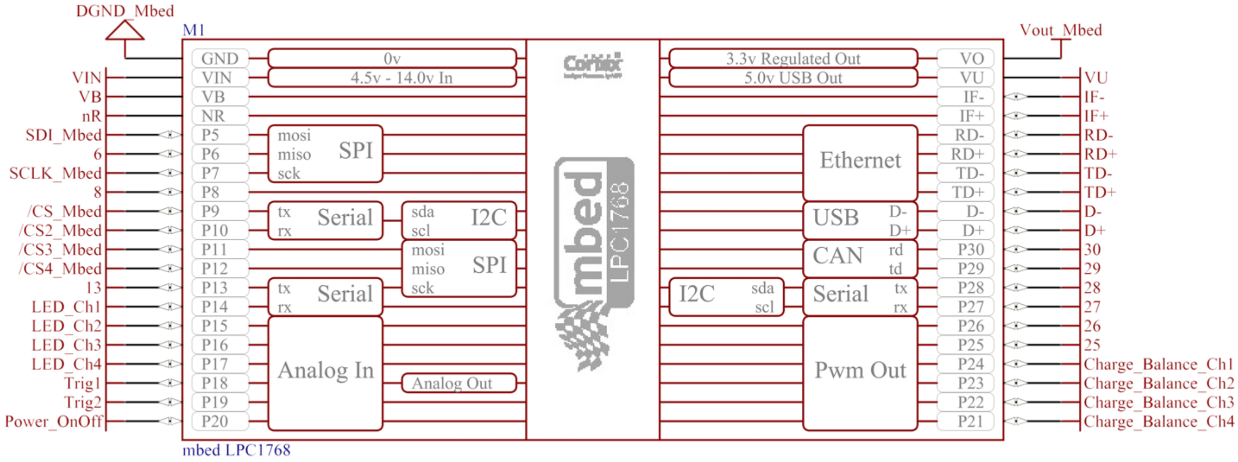

Optical switches

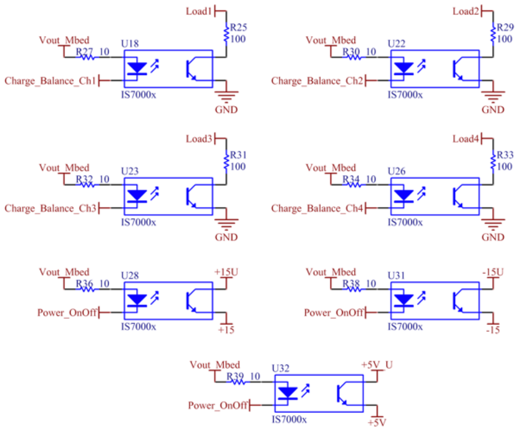

In/Out triggers

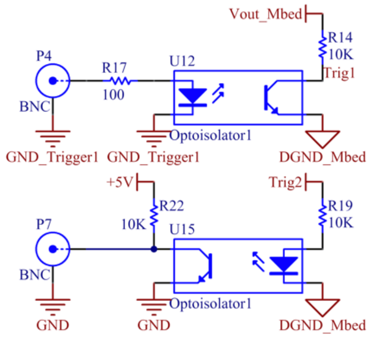

Channels LEDs

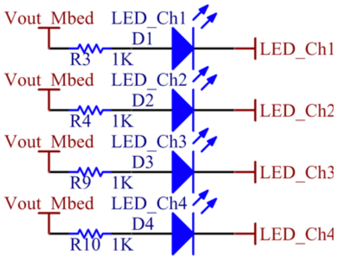

Stimulation Unit #1

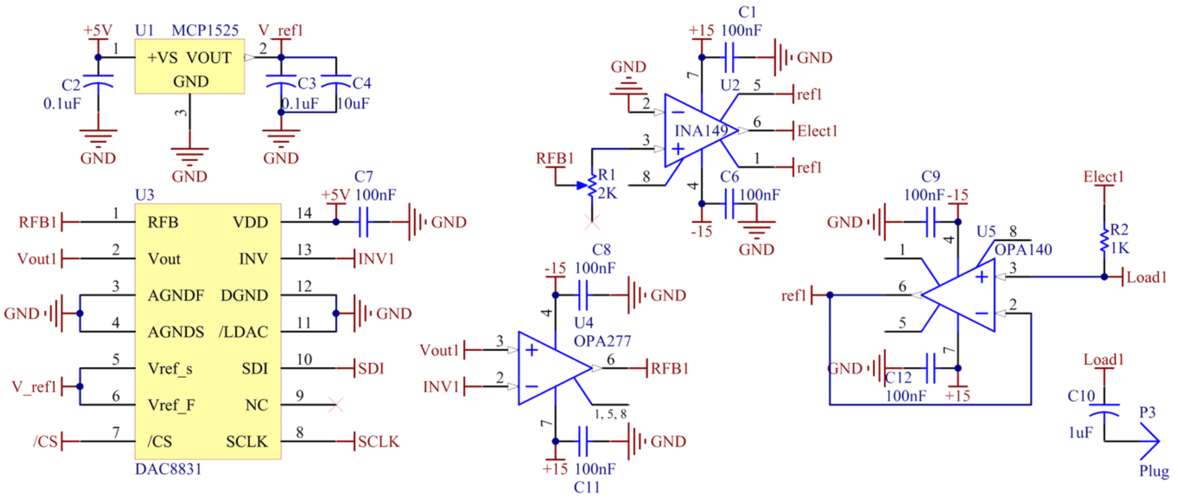

Stimulation Unit #4

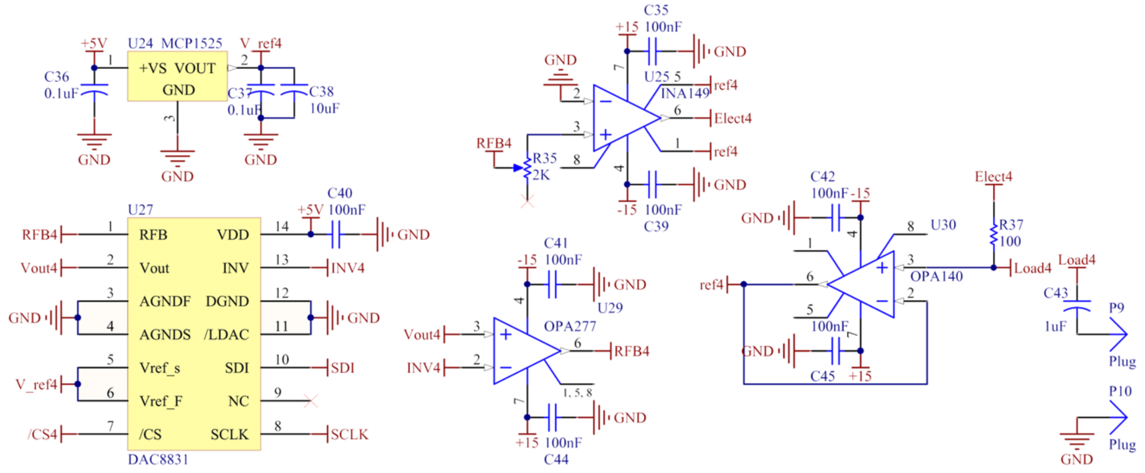

Part Name	Description	Qty. neede d	Order Code by Farnell	Unit Price	Total Price
OM11043	Cortex-M3 Board	1	1761179	40.20	40.2
DAC8831	16-bit DAC	4	2496196	12.37	49.48
INA149	High speed Differential Amp.	4	1459431	6.72	26.88
OPA277	OP. Amp	4	1097478	2.73	10.92
OPA140	OP. Amp	4	1855117	3.95	15.8
AQY212GH	MOSFET Relay	6	2503697	4.37	26.22
MCP1525	Voltage Ref.	4	9758500	0.768	3.072
TEL 5-1223	DC/DC Conv.	1	1204972	14.63	14.63
TMA 1205D	DC/DC Conv.	1	1007522	3.58	3.58
BNX002-01	Filter	4	9526943	4.13	16.52
Connectors	Connectors, Plugs, BNC	14			20
Passive Components	Resistors, Capacitors, Fuse, Inductors, Diode	110			10
PCBs	By pcb-pool				158.34
**SUM**					**395.64**
